# Randomized controlled trial of computerized working memory training for Veterans with PTSD

**DOI:** 10.1016/j.jpsychires.2024.11.072

**Published:** 2024-11-29

**Authors:** J. Bomyea, M.M. Caudle, A.L. Bartolovich, A.N. Simmons, A.J. Jak, S. Golshan

**Affiliations:** a Center of Excellence for Stress and Mental Health, VA San Diego Healthcare System, USA; b Department of Psychiatry, University of California San Diego, USA; c SDSU/UC San Diego Joint Doctoral Program in Clinical Psychology, USA; d VA San Diego Healthcare System, USA

**Keywords:** Cognitive training, Working memory, Executive functioning, Trauma, Posttraumatic stress disorder

## Abstract

Posttraumatic stress disorder (PTSD) is a common psychiatric condition among Veterans that is associated with deficits across a range of neuropsychological domains including working memory. While gold-standard psychosocial treatments are highly effective, there still remains a high rate of individuals who do not engage with or fully benefit from them. Cognitive training targeting specific working memory deficits in PTSD presents an alternative treatment approach that has shown promise for reducing re-experiencing symptoms. The current study evaluated a 16-session working memory training (WMT) program in Veterans with PTSD, focusing on two levels of interference control training. Feasibility, acceptability, and clinical efficacy were assessed. Results indicated that the intervention was generally feasible and acceptable to Veterans and revealed similar effects between groups in the intent to treat analyses; however, the high interference control training group yielded greater re-experiencing symptom reductions than the low interference control training group among individuals who were protocol adherent (Hedges’ *g* = 0.57). There were significant reductions in overall PTSD severity across groups. Results are broadly consistent with theoretical models and prior clinical trials linking working memory task improvement to re-experiencing symptom reductions. These results point to the potential for working memory training to be a viable intervention for PTSD in Veterans, though further research is necessary for validation and exploration of broader clinical outcomes.

## 1. Introduction

Posttraumatic stress disorder (PTSD) is characterized by an onset of symptoms in response to an exposure to at least one traumatic event (American Psychiatric Association, 2013). It is a relatively common psychiatric disorder among Veterans in the United States, occurring in approximately 9% of Veterans (Wisco et al., 2022). Evidence-based therapies such as prolonged exposure (PE) and cognitive processing therapy (CPT) can effectively reduce symptoms of PTSD if treatment is completed (Rutt et al., 2018), but these interventions are not universally effective. Despite high efficacy on average, up to two thirds of Veterans do not achieve full clinical remission with standard evidence-based treatments (Steenkamp et al., 2015). In addition, not all Veterans are fully willing or able to engage in a full course of treatment. One study found that, among Veterans diagnosed with PTSD, up to 55.8% dropped out of PE and up to 46.6% dropped out of CPT (Schnurr et al., 2022). Reasons offered by Veterans for early treatment termination include believing that they had worsened symptoms after PE as well as poor feasibility due to appointments interfering with other commitments, such as school, work, or family (Hundt et al., 2020; Wells et al., 2023). Thus, there continues to be a need to explore alternative treatment approaches, including those that can address symptoms via alternate administration formats beyond traditional psychotherapies.

Cognitive training, which involves practicing computerized tasks designed to engage specific thinking skills, may offer an alternative treatment approach for PTSD. Cognitive training programs seek to achieve clinical benefits by targeting the specific thinking skills thought to be involved in the onset or persistence of symptoms. Individuals with PTSD demonstrate impairments across a range of cognitive domains spanning verbal learning and memory, attention, and components of executive functioning, including working memory (Aupperle et al., 2012; Clausen et al., 2019; Moore, 2009; Scott et al., 2015; Stricker et al., 2015). These cognitive deficits impede the ability to engage top-down attention control, regulate memory retrieval, and disengage from negative thoughts and memories, each of which may contribute to the etiology and maintenance of PTSD symptoms (e.g., concentration difficulty, hypervigilance for threatening cues, and reliving trauma memories) (Aupperle et al., 2012; Jak et al., 2018). Cognitive training programs targeting these diverse neuropsychological processes have been tested as clinical interventions (e.g., attention control training, multifaceted cognitive-affective training, and response inhibition training), results of which demonstrate beneficial effects on PTSD symptoms (Badura-Brack et al., 2015; Echiverri-Cohen et al., 2021; Fonzo et al., 2019). The promising symptom effects observed in these trials, coupled with their relatively low burden and cost-effective nature, support continued investigation of cognitive training as a novel intervention for PTSD.

Working memory may be a particularly helpful training target given links between working memory and regulation of negative thoughts and memories. Working memory is a capacity limited store critical for holding information for processing that requires both selecting relevant information for processing and preventing irrelevant cues from intruding (Baddeley, 2012; Cowan, 2017). This latter component of preventing irrelevant information from occupying conscious thought–also referred to as control over working memory interference–is a critical component of overall working memory performance (Unsworth, 2010; Whitney et al., 2001). Recurrent negative thinking patterns like re-experiencing symptoms can be conceptualized as a type of interference, whereby trauma-related thoughts and memories intrude into active working memory storage and interfere with goal-directed cognitive activity (Aupperle et al., 2012; Bomyea and Lang, 2015; Gillie and Thayer, 2014; Levy and Anderson, 2008). Individuals with relatively poor working memory skills may be particularly prone to poor regulation of trauma-related thoughts and thus likely to endorse greater re-experiencing symptoms in the context of traumatic stress exposure. Experimental studies support this proposal by demonstrating associations between performance on cognitive paradigms requiring working memory interference control and greater intrusive memories in unselected samples completing in-lab stressors (Verwoerd et al., 2009; Wessel et al., 2010), intrusions during thought suppression tasks (Brewin and Beaton, 2002; Brewin and Smart, 2005), and re-experiencing symptoms in individuals with trauma exposure (Bomyea et al., 2012). Taken together, these studies also raise the possibility that improving regulation of interference in working memory could improve control over trauma-related thoughts and memories, thus reducing re-experiencing symptoms in PTSD.

Training programs targeting working memory have shown initial promise for reducing re-experiencing symptoms (Larsen et al., 2019). Given the proposed importance of interference control for re-experiencing symptoms of PTSD, Bomyea and colleagues (Bomyea and Amir, 2011; Bomyea et al., 2015) conducted two studies testing the effects of a training that specifically targeted working memory interference control on intrusive memories. In each study, one group was assigned to complete a working memory training program that had relatively high interference demands (i.e., highly similar stimuli to be remembered while solving puzzles), such that individuals repeatedly practiced engaging control over working memory interference. The other group completed a program with the same capacity demands for number of items to be remembered, but had relatively lower interference demands (i.e., dissimilar stimuli to be remembered while solving puzzles). In a single-session experimental study with unselected participants, completing the high versus low interference WMT was associated with less frequent intrusive memories about an ideographically selected negative autobiographical event during a thought suppression paradigm (Bomyea and Amir, 2011). In a second study using a multi-session clinical trial design, women with PTSD secondary to sexual trauma randomized to high interference WMT showed reductions in PTSD re-experiencing symptoms relative to those who completed low interference WMT, though participants in both groups showed reductions in global measures of PTSD severity (*d* = 0.52 – 0.92) (Bomyea et al., 2015). This trial demonstrated feasibility and acceptability of the WMT in a civilian group (i.e., participants rated the treatment as moderately logical and moderate confidence that it would be helpful, and there were no differences in number of sessions completed between groups) (Bomyea et al., 2015). These data point to the potential clinical utility of this type of intervention, but additional replication is needed to further evaluate clinical effects and explore outcomes in other samples with PTSD, including Veterans.

The current study evaluated the feasibility and preliminary clinical effects of WMT targeting working memory interference control in a sample of Veterans with PTSD [NCT03316196]. We randomized participants to complete a 16-session version of WMT with high or low interference control demands. As this is the first trial using this type of training program in a sample of Veterans with PTSD, we examined indicators of feasibility and acceptability based on participation and Veteran ratings of the program. In addition, we assessed the extent to which there was evidence of clinical efficacy. Based on prior evidence that WMT impacts re-experiencing symptoms (Bomyea et al., 2015; Larsen et al., 2019), we hypothesized that individuals randomized to high interference control WMT would show significantly greater reductions on this symptom cluster than those in low interference control WMT. Measurement of global PTSD symptom change was also included and was considered exploratory.

## 2. Methods

### 2.1. Participants

A total of 130 Veterans were recruited for potential participation in the study from the VA San Diego Healthcare System. Veterans contacted study personnel in response to 1) flyers posted in mental health and primary care clinics, and general community areas as authorized by the VA San Diego Healthcare System, 2) advertisements in print and web-based media in the general community, and 3) referrals from mental health and primary care clinics and research studies. Study inclusion criteria included 1) meeting primary current DSM-5 criteria for PTSD, 2) ages 21 to 55, 3) literacy in English, 4) intention to stay in the San Diego area for the duration of the study for completing in-person visits, and 5) willingness to attend assessment and treatment sessions. Exclusion criteria for the study included 1) lifetime history of psychotic disorders or bipolar disorder, 2) severe substance use disorder within the last year, 3) psychiatric conditions likely to adversely impact cognition and/or are deemed to require other primary psychological intervention, 4) a history of any neurological disorder that may be associated with cognitive dysfunction, including history of severe traumatic brain injury, 5) acute suicidality (defined as intent, plan and/or action for severe self-harm within the past 3 months), 6) presence of circumstances that require imminent intervention prior to other treatment (e.g., current domestic abuse), 7) plans for medication changes during the study timeframe, 8) current or planned evidence-based psychotherapy for PTSD within the study timeframe, 9) plans for changes to other psychosocial therapy within the study timeframe, and 10) presence of life-threatening or unstable medical conditions. Veterans who followed VA standard of care medication guidelines while taking psychotropic medications and those receiving psychosocial treatment for non-PTSD conditions were included in this study as long as they met a 6-week stability criterion.

### 2.2. Procedure

Veterans who were interested in the study completed a telephone screen to assess initial eligibility based on inclusion and exclusion criteria. All participants completed a baseline intake appointment where they provided informed written consent and completed initial clinical interviews and self-report measures. The Clinician-Administered PTSD Scale for DSM-5 (Weathers et al., 2013), the Mini-International Neuropsychiatric Interview (Sheehan et al., 1998), and interview questions regarding treatment and medical history were used to evaluate eligibility criteria. Following completion of baseline assessments, participants were randomized 1:1 to 16 sessions of high interference control WMT (HIC) or low interference control WMT (LIC). Randomization followed a randomly permuted block design and stratified by concurrent treatment status (none, medication, psychotherapy for non-PTSD conditions, or both) and age (dichotomized <36 or ≥ 36). Treatment assignment was maintained by the study statistician and blinded for the PI, research staff administering assessments and training, and participants. A brief set of self-report questionnaires was conducted at the mid-treatment timepoint for clinical monitoring purposes. After the training phase, participants completed post-training assessment visits where they repeated the pre-training assessments and completed a brief interview to assess changes to psychotherapy or medication during the study protocol. Two months post-treatment, participants completed a follow-up assessment with self-report questionnaires and interviews. Participants received monetary compensation of up to $300 for completing all study assessments. The trial protocol was approved by the VA San Diego Institutional Review Board. See supplementary materials (S1) for deviations from procedures specified in the trial registration. Notably, the COVID-19 pandemic occurred at the midpoint of this trial which resulted in changes in administration format, from in-person to remotely collected informed consent, interviews, questionnaires and treatment delivery using VA approved telehealth communication. Following the onset of COVID-19, all participants were given the option to complete all procedures remotely to accommodate participant preferences and COVID-19 guidelines.

### 2.3. Outcome measures

#### Clinician-Administered PTSD Scale for DSM-5 (CAPS-5).

The Clinician-Administered PTSD Scale for DSM-5 (Weathers et al., 2013) assesses PTSD diagnostic status as well as severity of symptoms using a semi-structured interview approach. The CAPS-5 was given at pre-treatment, post-treatment, and follow-up assessments. The re-experiencing cluster total was considered the primary outcome. Secondary outcomes from the CAPS-5 included diagnostic status at post and total severity scores.

#### The PTSD Checklist for DSM-5 (PCL-5).

The PTSD Checklist for DSM-5 (Weathers et al., 2013) was administered to measure PTSD symptom severity. The PCL-5 was given at pre-treatment, mid-treatment, post-treatment, and at follow-up assessments. The re-experiencing cluster total score and overall total scores were considered exploratory outcome measures to assess change in PTSD symptoms.

#### Treatment acceptability ratings.

The Therapy Evaluation Form (adapted from Holt and Heimberg, 1990) was administered to measure satisfaction and acceptability of the intervention. Items assessed how logical participants found the training programs, how successful they believed the training programs would be in addressing PTSD, and their confidence in recommending the training programs to other Veterans, rated on a 9-point scale ranging from 0 to 8.

### 2.4. Intervention

Participants were randomly assigned to a high interference control WMT condition (HIC) or low interference control WMT condition (LIC). Both HIC and LIC included 16 sessions completed over eight weeks (twice weekly), scheduled as 30-min appointments. Each appointment was facilitated by a research coordinator who established the session on telehealth technology and initiated the program, then recused themselves while the program was completed. The WMT was administered using E-Prime software (Psychology Software Tools, Inc) on a mouse-operated computer and consisted of a modified complex reading span task, which required the participant to remember item stimuli presented while doing a secondary problem solving or processing task. All stimuli presented were affectively neutral. Words and sentences were drawn randomly from a different set during each week of training. At the start of each trial, a fixation cross would appear for 500ms. Next, the screen displayed a processing task (e.g., “Jane walks her car in the park”). After the processing task was viewed, the screen displayed a question about the task and prompted the participant to mark an answer (the trial timed out after 5 s if no response was provided, which was considered an error). The processing task was then followed by the presentation of an item for 500ms. The subsequent trials followed the same pattern–the presentation of a processing task followed by an item. Once the participant reached the end of a set, they were shown a recognition screen with twelve items and were instructed to choose and arrange previously displayed items in serial order. After the recognition task, new trials began and followed the same pattern. The two conditions differed only on the amount of interference control required to successfully remember stimuli on each trial (for additional details see (Bomyea et al., 2015)). If a participant was randomized to HIC, they completed trials that required high interference control. All to-be-remembered items were derived from the same category (e.g., all letter stimuli), based on prior data suggesting that maintaining the same type of to-be-remembered items across trials maximizes interference (Bunting, 2006). By requiring repeated practice with utilization of interference control across trials, HIC was thought to enhance plasticity of cognitive systems and improve performance. If a participant was randomized to LIC, they completed trials that required less interference control but the same amount of overall working memory capacity. Every three trials, all to-be-remembered items alternated categories (e.g., letters, then numbers). Although the number of items that participants needed to remember was the same as HIC, LIC had less interference because the alternation of items based on category (letters and numbers) reduced the chance of interference across trials. All participants, regardless of condition, completed 45 self-paced trials at each visit, with each visit lasting approximately 30 min. The 45 trials were administered as 3 equivalent runs and all participants were given the opportunity to take short breaks between each run. Accuracy on the training tasks was calculated as the total number of items recalled correctly in the correct serial position.

### 2.5. Statistical analyses

Comparison of baseline differences was conducted using analysis of variance (ANOVA) or *χ*^2^ as appropriate. Examination of feasibility was conducted by examining descriptive statistics of number of completed sessions, self-report ratings of acceptability, and descriptive statistics on participants who showed clinical deterioration (>20% increase in symptoms from pre-to post-training and number of significant adverse events). Intent to treat (ITT) analysis of symptom change was conducted with all randomized participants through linear mixed effects models using restricted maximum likelihood (REML) with a random effect of subject modeled. The analyses used a mixed design with one grouping factor (HIC, LIC) and one within factor of time with two levels for the CAPS-5 (baseline, post) and three levels for the PCL-5 (baseline, mid-treatment [after session 8], post). Models predicted each outcome separately using fixed effects of group, time, and group by time interactions, controlling for stratification variables with time centered at the post-training timepoint. We also conducted a supplemental per protocol (PP) analysis where individuals were considered missing from the point at which they because no longer adherent to the protocol as intended (n = 22 total participants). Protocol adherence was defined by 1) completing a minimum of 4 treatment sessions consistent with prior work (n = 10; HIC = 5 LIC = 5 (Bomyea et al., 2015);), and 2) maintaining stability of other treatments, if received, for the duration of the study (n = 13; HIC = 6 LIC = 7 changed evidence-based treatment for PTSD symptoms during training). The PP analysis used the same analytic steps as the ITT, but individuals’ data were censored starting from the timepoint where they stopped protocol adherence. Primary analyses focused on pre-post data consistent with the study registration (a full report of all CAPS-5 and PCL-5 cluster scores can be found in the Supplemental Materials). In cases where the outcome category had multiple assessments (i.e., secondary outcomes (2) and exploratory outcomes (2)) FDR correction was used; all effects surviving multiple correction are specified in the results tables. We conducted an additional exploratory analysis including pre-, mid-, and post-treatment, and 2-month follow-up time points to explore the effects of time and treatment condition at follow-up (see Supplemental Materials S2–S3). Estimates of effect sizes for clinical outcomes were calculated using between group Hedges *g* at posttreatment. Finally, linear mixed effects models were also used in a post-hoc exploratory analysis examining relationships between training-related changes in working memory performance (i.e., total accuracy in training sessions) and change in symptoms, using calculated difference scores between pre and post re-experiencing symptom measures.

To determine the sample size, power calculations were conducted prior to beginning the study using the *longpower* package in R with effect size estimates derived from the prior randomized controlled trial of this intervention in women with PTSD for CAPS outcomes (*f* = 0.25). Because effect sizes from prior pilot studies could overestimate the effect size in Veterans, either due to differences in population characteristics (e.g., greater severity, sociodemographic characteristics) or greater attrition, linear mixed effects power calculations were conducted for a range of smaller effect sizes and smaller sample sizes to ensure that the proposed sample would be sufficiently powered to test the primary outcome. Data simulations using the powerSim package in R estimated power and confidence intervals for power estimates (bootstrapped with 1000 iterations) for a range of effect sizes and sample sizes suggested that sufficient power (i.e., >80%) would be attained with the proposed sample across a range of more modest effect sizes (i.e., a small to medium effect) and higher attrition rates (up to 40%). All remaining analyses were conducted in SPSS version 23.

## 3. Results

### 3.1. Clinical and demographic characteristics

**Fig. 1.**
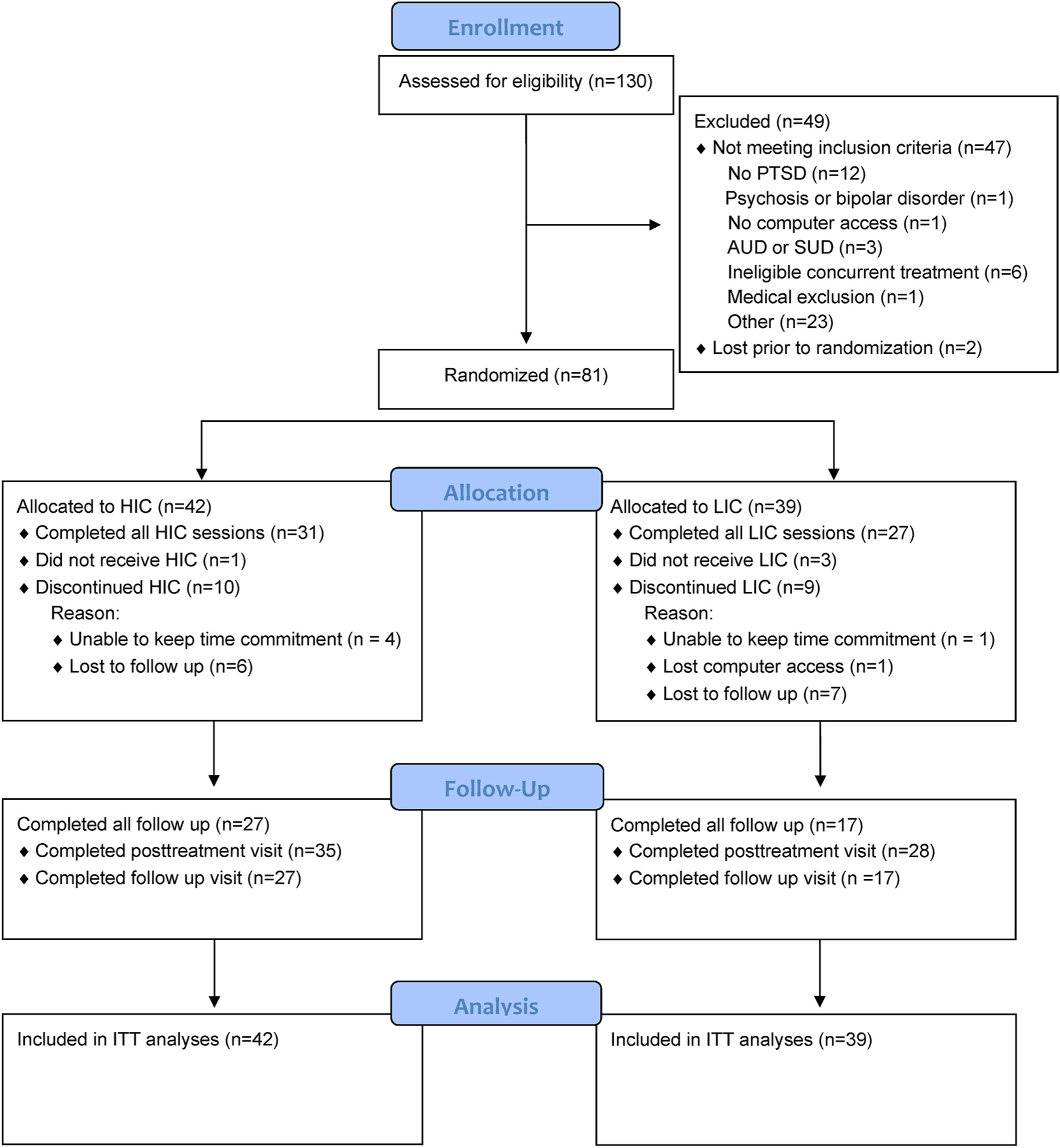
CONSORT flow diagram summarizing participant progress throughout the study.

The final sample included 81 participants randomized to LIC or HIC (see CONSORT diagram in Fig. 1). There were no statistically significant differences in sociodemographic or clinical characteristics between HIC and LIC groups (*ps* > 0.08; see Table 1). The most common comorbid diagnoses included major depressive disorder (n = 34; LIC = 15, HIC = 19), anxiety disorders (n = 60; LIC = 27, HIC = 33), and alcohol or substance use disorder (n = 13; LIC = 9, HIC = 14). There were no statistically significant differences between groups on baseline symptom severity as measured by the CAPS-5, *F*(1,79) = 0.02, *p* = .89, or the PCL-5, *F*(1,79) = 0.05, *p* = .82. There were also no statistically significant differences in baseline working memory, as measured by performance during the initial session *F*(1,76) = 0.62, *p* = .43. There were no statistically significant associations between protocol adherence status (total adherent LIC n = 27, HIC n = 31) and sociodemographic or baseline PTSD symptom severity scores (*p*s > 0.2).

### 3.2. Feasibility and acceptability

Out of 81 Veterans who were randomized, 78 (96.3%) attended at least one session (LIC = 36; HIC = 41) and 58 (71.6%) completed all sessions (LIC = 27; HIC = 31). There was no statistically significant difference between the number of Veterans completing all sessions between the HIC and LIC groups, χ^2^(1) = 0.21, *p* = .65. On a 9-point scale, participants endorsed moderate ratings on perceptions of how logical the intervention seemed (*M(SD)* = 4.59(2.32)), confidence in treatment efficacy (*M(SD)* = 5.32(1.88)), and confidence in recommending treatment to others (*M(SD)* = 4.78(2.31)) with no differences observed between groups (*p*s > 0.44). Two participants demonstrated clinical deterioration (2.5% of those who received treatment; LIC = 1, HIC = 1), and no significant adverse events were reported.

### 3.3. Primary outcome: CAPS re-experiencing symptoms

Individuals randomized to HIC did not demonstrate differential reductions in CAPS-5 re-experiencing symptom severity in comparison with those assigned to LIC (see Table 2a–b for scores by group and effect sizes). Across both groups, CAPS-5 re-experiencing symptoms showed statistically significant reductions over time. In contrast, in the PP analyses individuals in the HIC group showed a significantly greater reduction in CAPS-5 re-experiencing symptoms than those in the LIC group.

### 3.4. Secondary outcome: CAPS-5 diagnostic status and total score

Among those completing the post-treatment diagnostic interview, 42.9% of individuals in the LIC group and 40% of individuals in the HIC group no longer met diagnostic criteria for PTSD. There were no statistically significant differences in CAPS-5 diagnosis at post-treatment in LIC versus HIC in ITT analyses, χ^2^(1) = 0.05, *p* = .81, or PP samples, χ^2^(1) = 0.42, *p* = .52. There was a statistically significant reduction in CAPS-5 total symptom severity over time in both the ITT and PP analyses. Individuals randomized to HIC did not demonstrate greater reductions in CAPS-5 total symptom severity in comparison with those assigned to LIC in the ITT or PP samples (Table 2c–d).

### 3.5. Exploratory outcomes: PCL-5 re-experiencing and total score

Individuals randomized to HIC did not demonstrate differential reductions in PCL-5 re-experiencing symptom severity in comparison with those assigned to LIC in ITT analyses. Across both groups, PCL-5 re-experiencing symptoms showed statistically significant reductions over time. In contrast, in the PP analyses, individuals in the HIC group showed a greater reduction in PCL-5 re-experiencing symptoms than those in the LIC group (Table 2e–f), though results were not significant after accounting for multiple comparison correction based on the two exploratory outcomes analyzed. Statistically significant reductions in PCL-5 total severity scores were observed across groups in both ITT and PP analyses, but effects of group on PCL-5 total severity scores did not reach statistical significance (Table 2g–h).

### 3.6. Exploratory analysis: change in WM during training and relationship to change in re-experiencing symptoms

As an exploratory post-hoc analysis, we examined the relationship between trajectory of improvement in working memory task performance during the training sessions (smoothed by averaging the sessions completed biweekly) and pre-to post-training difference in re-experiencing symptoms. The baseline model examining change over time by group revealed significant improvements in performance over time, β = 7.75, SE = 2.87, *p* = .008; no statistically significant moderating effect of group was observed, β = −0.16, SE = 1.74, *p* = .93 (initial month performance in LIC: M = 67.3%(21.5%) HIC: M = 63.2%(24.6%); final month performance in LIC: M = 74.2%(24.0%), HIC: M = 75.1% (27.5%)). Results of the longitudinal models relating change in working memory task performance to change in re-experiencing symptoms on the CAPS-5 did not reveal a significant relationship, β = −0.13, SE = 0.31, *p* = .66. However, results of the longitudinal models relating change in working memory task performance to change in re-experiencing symptoms on the PCL-5 revealed a statistically significant effect, β = 0.60, SE = 0.22, *p* = .006. Individuals with greater improvement in PCL-5 re-experiencing symptoms showed greater improvement in working memory task performance.

## 4. Discussion

The current study sought to examine clinical effects of WMT in Veterans with PTSD. We found that Veterans generally rated the program favorably, adherence was high, and there were no adverse events or significant evidence of clinical deterioration. Contrary to the hypothesis, reductions in CAPS-5 re-experiencing symptoms did not differ between the HIC and LIC groups in the ITT analyses; both acute treatment effects and exploratory analyses over the follow up interval showed reductions over time. However, in the PP analyses there were greater reductions in CAPS-5 re-experiencing symptoms among the HIC group. After WMT, there was a decrease in the number of individuals who met CAPS-5 diagnostic criteria for PTSD and a reduction in CAPS-5 total symptom severity in both the HIC and LIC groups. In addition, reductions in PCL-5 re-experiencing symptom severity did not differ between the two groups in the ITT analyses; however, in the PP analyses, there were higher reductions in PCL-5 re-experiencing symptom severity among the HIC group. Post-hoc analyses revealed that individuals who had more improvement in PCL-5 re-experiencing symptoms had more improvement in working memory task performance. There was evidence from both our secondary outcome and non-registered outcomes (described in the supplemental materials) that group effects were specific to re-experiencing related symptoms.

Results in the current study are broadly consistent with models linking working memory interference control to re-experiencing symptoms with important caveats. Re-experiencing symptoms are thought to stem from deficits in working memory ability needed to regulate the contents of thought generally (Anderson and Levy, 2009; Aupperle et al., 2012; Bomyea and Lang, 2015). Individuals with relatively poor working memory may be at risk for greater preponderance of trauma-related intrusive thoughts because they lack adequate cognitive resources to down-regulate irrelevant or unwanted thoughts and memories when prompted by trauma-relevant retrieval cues. Bolstering working memory ability through training could thus improve thought regulation, potentially reducing trauma-related thoughts and memories that drive re-experiencing symptoms. We found provisional support for the hypothesis that working memory training targeting interference control would be associated with re-experiencing symptoms, consistent with prior work using working memory training for PTSD (Bomyea et al., 2015; Larsen et al., 2019). When comparing the two training groups, there was a differential improvement in re-experiencing symptoms favoring those receiving the HIC with high interference demands compared to those receiving the LIC with low interference demands. However, these effects were limited to the analyses with protocol adherent participants. In the ITT and PP analyses of global measures of PTSD severity (i.e., CAPS-5 total scores and the percentage of individuals remitted from their PTSD diagnosis), groups showed reductions that were similar across groups. Two potential explanations for discrepancies between ITT and PP results fall under the umbrella of treatment compliance. First, while changes to concurrent pharmacotherapy or initiation of treatment for PTSD was considered exclusionary, a number of participants initiated new pharmacological or evidence-based treatment for PTSD while enrolled. We did not withdraw these individuals from receiving WMT for ethical reasons, but their inclusion impacts the interpretation of the ITT analysis (e.g., individuals initiating new evidence-based treatments alongside our planned sham could have led to symptom improvement that was unrelated to their assigned condition in the protocol). Second, treatment effects are anticipated to be higher in those who receive an adequate amount of training sessions. Accounting for these factors in the PP analyses by focusing analyses only on those participants considered compliant to the protocol produced more robust treatment effects that can better differentiate the relatively minor nuance between HIC and LIC conditions. Despite these potential explanations, PP analyses must be interpreted with great caution. While PP analyses have advantages in terms of knowing how an intervention administered “purely” under optimal conditions, it also loses key advantages of randomization (Smith et al., 2021) and may introduce bias as it favors those who adhere to treatment, potentially limiting generalizability. In contrast, ITT emphasizes treatment effectiveness, reflecting real-world scenarios where not all patients adhere to the intervention, helping to maintain original randomization and minimize bias, thus providing a more realistic estimate of treatment effects in the general population.

Improvements in working memory task performance were observed in both groups. There are a number of reasons why working memory task performance may not have differed by group assignment. First, both groups completed relatively rigorous cognitive activities and likely benefitted from repeated practice. The metrics derived from the two groups from training tasks were necessarily different – by design, the LIC group was “easier” than the HIC group based on the interference demands, and thus performance metrics across the two groups were not fully equivalent. In addition, both groups completed up to 16 sessions. We selected the higher number of training sessions (16 versus 8 sessions completed in prior work) to provide a strong “dose” to our Veteran sample, which may be somewhat more clinically complex than the individuals in the initial pilot sample. It is possible that participants in both groups exceeded the threshold of amount of practice needed for improvements, resulting in a ceiling effect. Across groups, there was an association between improvements in working memory task performance and improved re-experiencing symptoms. Consistent with an experimental medicine approach (Insel, 2015; Zucker et al., 2023), this suggests a potential link between the treatment target (working memory task performance) and clinical outcomes (PTSD symptoms) that was observed irrespective of the subtle interference manipulation across HIC and LIC. Future studies should include a more comprehensive assessment battery to measure cognitive effects, particularly generalization to novel tasks and cognitive processes, that would support meaningful transfer beyond simple practice effects.

Results should be interpreted in light of several limitations. The design of the study did not include a waitlist or non-training condition that did not involve a computer interface and working memory demand. The control condition was selected with the intention of isolating the interference control component of training, but resulted in two groups that received a relatively high degree of training on non-interference control elements of working memory. Observations of similar reductions in overall symptoms (e.g., reduced total scores, reduced symptom cluster scores aside from re-experiencing) could be attributed to these training elements, but future work will be needed to determine the extent of changes attributable to these components. For example, future studies might benefit from a control condition that involves low working memory demand based on only testing small set sizes, or a repeated assessment format that requires equivalent time with an experimenter but no computerized interactions. This would be particularly helpful for parsing out the extent to which any general effects of PTSD symptom reduction were due to elements of shared by both the HIC/LIC interventions. Working memory was only assessed through one task in the study, which limits the ability to determine if improvements in working memory task performance were related to actual improvements in cognitive functioning. Future studies should include additional assessments of cognition in order to determine if cognitive functioning improved. The sample included outpatient individuals with relatively mild overall severity and was primarily male OEF/OIF era. Thus, future work is needed to examine generalizability of effects to a broader range of Veterans. While concurrent intervention type was included as a stratification factor, we did not collect detailed information about number of treatment sessions or modality to explore relationships between these features and clinical response. We did not select individuals based on evidence of pre-existing neurocognitive deficits. Tailoring the intervention to individuals presenting with cognitive complaints could provide a more efficient approach for testing clinical effects. The study had provisional evidence that training effects persisted, however the follow up interval was relative short, and effects were specific to interview rated scores, thus future studies should more comprehensively evaluate the longevity of any observed effects. Finally, the COVID-19 pandemic occurred at the midpoint of this trial. As a result, there were changes in administration format to remotely delivered treatment and some individuals enrolled in the trial experienced an acute exacerbation of health and economic stressors. We were underpowered to fully test potential effects of the pandemic, underscoring the need for future replication of the trial.

In summary, the current data suggest that WMT in Veterans is feasible and well tolerated and provide provisional evidence that WMT programs targeting interference control may impact re-experiencing symptoms. Training interventions with and without strong interference demands resulted in practice-related improvements, and the extent to which individuals improved related to their improvement on self-reported re-experiencing symptom severity. It is plausible that working memory training programs offer a treatment approach that directly addresses cognitive mechanisms of re-experiencing symptoms, which may be complementary to traditional evidence-based practices that focus on the emotional content of trauma-related thoughts. Future studies are needed to evaluate efficacy and understand how WMT can be implemented clinically alone or in concert with other treatments in a controlled way. If found to be efficacious, WMT may be a low burden and low risk, cost effective, and easy to disseminate tool for reducing PTSD symptoms.

## Supplementary Material

1

Appendix A. Supplementary data

Supplementary data to this article can be found online at https://doi.org/10.1016/j.jpsychires.2024.11.072.

## Figures and Tables

**Table 1:** Sociodemographic and clinical characteristics.

Characteristic	LIC (n = 39)	HIC (n = 42)	Statistic (LIC versus HIC)
Sex n (% male)	24(61.54)	29(69.05)	χ^2^(1) = 0.5, *p* = .49
Age in years M(SD)	37.77(8.21)	37.57(8.53)	*F*(1,78) < 0.01, *p* = .98
Education in years M(SD)	15.62(2.25)	15.24(2.62)	*F*(1,78) = 0.46, *p* = .50
Race n (%)			χ^2^(8) = 14.04, *p* = .08
Black	4(10.26)	3(7.14)	
White	20(51.28)	29(69.05)	
Asian	5(12.82)	0(0)	
Native American/Alaskan Native	2(5.13)	2(4.76)	
Native Hawaiian/Pacific Islander	3(7.69)	0(0)	
Biracial	2(5.13)	1(2.38)	
Not reported	3(7.69)	7(16.67)	
Hispanic ethnicity n (%)	12(30.77)	17(40.48)	χ^2^(1) = 0.83, *p* = .49
Baseline working memory capacity accuracy percentage	61.7(23)	65.8(22)	*F*(1,76) = 0.62, *p* = .43

**Table 2:** Clinical effect over time in ITT and PP analyses.

Measure (*Range*)	Baseline *M(SD)*	Post-training Assessment *M(SD)*	ES post-Treatment Hedges’ *g*	Mixed model effects Beta (SE), statistic and p-value	
**a. CAPS-5 re-experiencing symptom severity ITT *(0***–***20)***				Time:	β = 2.03(0.59), *t* = 3.46, *p* = .001
				Group^a^ Time:	β = 0.02(0.36), *t* = 0.04, *p* = .97
LIC	8.77(3.06)	6.46(3.98)	0.02		
HIC	8.90(2.56)	6.40(3.77)			
**b. CAPS-5 re-experiencing symptom severity PP *(0***–***20)***				Time:	β = 2.51(0.77), *t* = 3.25, *p* = .002
				Group^a^ Time:	β = −0.96(0.40), *t* = −2.4, *p* = .02
LIC	8.77(3.06)	7.94(3.28)	0.57		
HIC	8.90(2.56)	5.85(3.81)			
**c. CAPS-5 total symptom severity ITT *(0***–***80)***				Time:	β = 6.53(2.00), *t* = 3.26, *p* = .002^a^
				Group^a^ Time:	β = 0.96(1.25), *t* = 0.77, *p* = .44
LIC	38.00(9.75)	26.29(14.00)	0.15		
HIC	37.88(9.15)	28.34(13.60)			
**d. CAPS-5 total symptom severity PP *(0***–***80)***				Time:	β = 7.18(2.69), *t* = 2.67, *p* = .01^a^
				Group^a^ Time:	β = −1.42(1.4), *t* = −1.01, *p* = .32
LIC	38.00(9.75)	29.78(12.55)	0.20		
HIC	37.88(9.15)	26.96(14.08)			
**e. PCL-5 re-experiencing symptom severity ITT *(0***–***20)***				Time:	β = 1.66(0.81), *t* = 2.04, *p* = .04
				Group^a^ Time:	β = −0.36(0.50), *t* = −0.72, *p* = .47
LIC	10.79(4.18)	10.07(5.75)	0.05		
HIC	11.45(4.29)	9.83(4.70)			
**f. PCL-5 re-experiencing symptom severity PP *(0***–***20)***				Time:	β = 1.97(1), *t* = 1.97, *p* = .05
				Group^a^ Time:	β = (0.54), *t* = −2.12, *p* = .04
LIC	10.79(4.18)	11.57(4.99)	0.36		
HIC	11.45(4.29)	9.82(4.70)			
**g. PCL-5 total symptom severity ITT *(0***–***80)***				Time:	β = 7.29(2.62), *t* = 2.79, *p* = .01^a^
				Group^a^ Time:	β = −0.58(1.63), *t* = −0.36, *p* = . 72
LIC	46.32(15.15)	40.25(19.36)	0.01		
HIC	47.10(14.80)	40.11(18.78)			
**h. PCL-5 total symptom severity PP *(0***–***80)***				Time:	β = 9.34(3.22), *t* = 2.9, *p* = .004^a^
				Group^a^ Time:	β = −2.32(1.72), *t* = −1.35, *p* = .18
LIC	46.32(15.15)	44.19(17.18)	0.01		
HIC	47.10(14.80)	38.28(18.68)			

a Designates effects that survive FDR multiple correction.
