# Supplementary material for: Randomized controlled trial of computerized working memory training for Veterans with PTSD
Source: J Psychiatr Res. Author manuscript; Available in PMC 2025 Sep 11. (PMC12425496; doi:10.1016/j.jpsychires.2024.11.072)
Supplement: 1 [file NIHMS2105687-supplement-1.docx]

**Randomized controlled trial of computerized working memory training for Veterans with PTSD**

Bomyea, J.^1-2*^, Caudle, M. M. ^3^, Bartolovich, A.^2^, Simmons, A. N. ^1-2^, Jak, A. J. ^1-2^, Golshan, S.^2-4^

^1^ Center of Excellence for Stress and Mental Health, VA San Diego Healthcare System

^2^ Department of Psychiatry, University of California San Diego

^3^ SDSU/UC San Diego Joint Doctoral Program in Clinical Psychology

^4^ VA San Diego Healthcare System

^*^ Corresponding author:

Jessica Bomyea, Ph.D.

9500 Gilman Drive (Mail code 0855)

# La Jolla, CA, 92093-0855, USA

Email : jbomyea@health.ucsd.edu

Requests for de-identified data, analysis code, and research materials should be made to Jessica Bomyea, [jbomyea@health.ucsd.edu](mailto:jbomyea@health.ucsd.edu)

Key words: cognitive training, working memory, executive functioning, trauma, posttraumatic stress disorder

**Supplementary Materials**

**S1. Deviations from trial registration**

Procedures were modified to minimize face-to-face contact during the COVID pandemic by transitioning assessments and training sessions to telehealth administration. As a result, a substantial portion of individuals did not complete the primary pencil-paper neuropsychological assessment stated in the trial registration. In lieu of this outcome measure, we report on cognitive performance during training session to provide a proxy for measuring cognitive improvement. We also report on an additional exploratory outcome of PCL reexperiencing symptoms. This assessment was added prior to onset of enrollment to provide complementary information to the interview-based primary outcome and was administered at the baseline, mid-treatment, post-training, and 2-month follow-up, thus providing potentially greater power to evaluate group effects. In June 2020 the inclusion criteria were expanded to include individuals with PTSD secondary to all trauma types, not just interpersonal trauma, to facilitate recruitment and increase generalizability.

**S2. Exploratory Outcomes: 2-month follow-up, CAPS-5 reexperiencing and total score**

We conducted additional ITT exploratory analyses including pre-, and post-treatment and 2-month follow-up time points to explore the effects of time, treatment condition, and their interaction at follow-up, controlling for stratification variables. The models revealed no differential reductions in CAPS-5 reexperiencing symptoms or total severity between the HIC and LIC conditions. Across both groups, the linear mixed effects models examining change over time by group revealed a significant improvement in in both CAPS-5 reexperiencing symptoms (*p* = 0.01) and CAPS-5 total severity score (*p* < .01) (Table S1a-b).

**S3. Exploratory Outcomes: 2-month follow-up, PCL-5 reexperiencing and total score**

We conducted additional ITT exploratory analyses including pre-, mid- and post-treatment, and 2-month follow-up time points to explore the effects of time, treatment condition, and their interaction at follow-up, controlling for stratification variables. No differential reductions in PCL-5 reexperiencing symptoms or PCL-5 total severity score were observed between individuals assigned to the HIC and LIC conditions. Across both groups, changes in PCL-5 reexperiencing symptoms and PCL-5 total severity scores over time did not reach significance (Table S1c-d).

**S4. Exploratory Outcomes: Avoidance, Arousal, and Negative Cognition and Mood symptoms on CAPS and PCL-5**

We conducted additional ITT and PP exploratory analyses on CAPS and PCL-5 avoidance, hyperarousal, and negative cognition and mood symptoms change to explore the effects of time, treatment condition, and their interaction (Table S2). The models revealed no statistically significant differential reductions in these symptom clusters between the HIC and LIC conditions. Across both groups, the linear mixed effects models examining change over time revealed significant ITT effects of time, such that on average participants showed reductions in avoidance, arousal, and negative cognition and mood symptoms.

Supplemental Table

Table S1

*Across both groups, CAPS-5 reexperiencing symptoms and CAPS-5 total symptom severity significantly improved across time.*

|  |  |  |  |  |  |  |  |  |  |
| --- | --- | --- | --- | --- | --- | --- | --- | --- | --- |
| Measure | | Baseline *M(SD)* | 2-Month Follow-up training Assessment *M(SD)* | ES 2-Month Follow-up -  Treatment  Hedges’ *g* | Mixed model effects  Beta (SE), statistic and p-value | | | | |
| a. CAPS-5 reexperiencing symptom severity ITT | | | |  | Time:  Group * Time: | | | | β = 1.15(0.43), t = 2.69, *p* = 0.01  β = -0.68(0.44), t = -1.54, *p* = 0.13 |
|  | LIC | 8.77(3.06) | 4.94(3.73) | 0.44 |  |  |  |  |  |
|  | HIC | 8.90(2.56) | 6.57(3.71) |  |  |  |  |  |  |
| b. CAPS-5 total symptom severity ITT | | | |  | Time:  Group * Time: | | | | β = 4.36(1.39), t = 3.15, *p* = 0.002  β = -1.95(1.44), t = -1.36, *p* = 0.18 |
|  | LIC | 38.00(9.75) | 22.12(13.14) | 0.44 |  |  |  |  |  |
|  | HIC | 37.88(9.15) | 27.96(13.73) |  |  |  |  |  |  |
| c. PCL-5 reexperiencing symptom severity ITT | | | | | Time:  Group * Time: | | | | β = 0.81(0.54), *t* = 1.50, *p* = 0.14  β = -0.77(0.56), *t* = -1.39, *p* = 0.17 |
|  | LIC | 10.79(4.18) | 7.76(4.56) | 0.59 |  |  |  |  |  |
|  | HIC | 11.45(4.29) | 10.29(4.11) |  |  |  |  |  |  |
| d. PCL-5 total symptom severity ITT | | | |  | Time:  Group * Time: | | | | Β = 3.03(1.80), t = 1.68, *p* = 0.09  β = -2.19(1.86), t = -1.18, *p* = 0.24 |
|  | LIC | 46.32(15.15) | 31.71(18.67) | 0.18 |  |  |  |  |  |
|  | HIC | 47.10(14.80) | 40.86(15.98) |  |  |  |  |  |  |

*Note*. Time was centered at 2-month follow-up visit.

Table S2

*Across both groups, CAPS-5 symptoms generally improved over time.*

|  |  |  |  |  |  |  |
| --- | --- | --- | --- | --- | --- | --- |
|  |  |  |  |  |  |  |
| **Measure** | | Baseline *M(SD)* | Post-training Assessment *M(SD)* | ES post- Treatment Hedges’ g | Mixed model effects Beta (SE), statistic and p-value | |
|  |  |  |  |  |  |  |
| **a. CAPS avoidance symptom severity ITT** | | | |  | Time: Group * Time: | β = 0.9(0.41), t = 2.21, *p* = 0.03 |
|  |  |  |  |  |  | β = 0.48(0.25)*, t* = 1.89, *p* = 0.06 |
|  | LIC | 4.33(1.84) | 2.46(2.12) | .45 |  |  |
|  | HIC | 4.38(1.56) | 3.43(2.12) |  |  |  |
| **b. CAPS avoidance symptom severity PP** | | | |  | Time: Group * Time: | β = 0.61(0.55)*, t* = 1.11, *p* = 0.27 |
|  |  |  |  |  |  | β = 0.2(0.29)*, t* = 0.71, *p* = 0.48 |
|  | LIC | 4.38(1.56) | 3.25(2.20) | .08 |  |  |
|  | HIC | 4.33(1.84) | 2.89(2.11) |  |  |  |
| **c. CAPS hyperarousal symptom severity ITT** | | | |  | Time: | β = 1.34(0.56)*, t* = 2.37, *p* = 0.021 |
|  |  |  |  |  | Group * Time: | β = 0.18(0.35),=0.52, *p* = 0.61 |
|  | LIC | 10.79(2.82) | 8.00(3.80) | .12 |  |  |
|  | HIC | 10.81(3.07) | 8.51(4.08) |  |  |  |
| **d. CAPS hyperarousal symptom severity PP** | | | |  | Time: | β = (0.83)*, t* = 1.31, *p* = 0.2 |
|  |  |  |  |  | Group * Time: | β = -0.34(0.43)*, t* = -0.79, *p* = 0.43 |
|  | LIC | 10.81(3.07) | 8.39(4.03) | .10 |  |  |
|  | HIC | 10.79(2.82) | 8.78(3.34) |  |  |  |
| **e. CAPS negative affect and cognitions symptom severity ITT** | | | |  | Time: | β = 2.3(1.04)*, t* = 2.22, *p* = 0.03 |
|  |  |  |  |  | Group * Time: | β = 0.27(0.64)*, t* = 0.43, *p* = 0.67 |
|  | LIC | 13.79(4.68) | 9.39(6.48) | .10 |  |  |
|  | HIC | 14.10(5.00) | 10.00(5.99) |  |  |  |
| **f. CAPS negative affect and cognitions symptom severity PP** | | | |  | Time: | β = 2.95(1.38)*, t* = 2.14, *p* = 0.036 |
|  |  |  |  |  | Group * Time: | β = -0.38(0.72)*, t* = -0.53, *p* = 0.6 |
|  | LIC | 13.79(4.68) | 9.46(5.83) | 0.06 |  |  |
|  | HIC | 14.10(5.00) | 10.22(6.25) |  |  |  |
| **g. PCL avoidance symptom severity ITT** | | | |  | Time: | β = 0.83(0.39)*, t* = 2.12, *p* = 0.04 |
|  |  |  |  |  | Group * Time: | β = 0.1(0.24)*, t* = 0.42, *p* = 0.67 |
|  | LIC | 5.42(2.09) | 4.29(2.46) | .02 |  |  |
|  | HIC | 5.38(1.95) | 4.57(2.10) |  |  |  |
| **h. PCL avoidance symptom severity PP** | | |  |  | Time: | β = 1.02(0.5)*, t* = 2.02, *p* = 0.05 |
|  |  |  |  |  | Group * Time: | β = -0.09(0.27)*, t* = -0.32, *p* = 0.75 |
|  | LIC | 5.00(2.21) | 4.43(2.17) | .11 |  |  |
|  | HIC | 5.42(2.09) | 4.67(2.33) |  |  |  |
| **i. PCL hyperarousal symptom severity ITT** | | | |  | Time: | β = 2.04(0.85)*, t* = 2.41, *p* = 0.02 |
|  |  |  |  |  | Group * Time: | β = -0.21(0.53)*, t* = -0.4, *p* = 0.69 |
|  | LIC | 14.12(4.55) | 11.71(5.50) | .13 |  |  |
|  | HIC | 13.55(4.45) | 12.06(6.09) |  |  |  |
| **j. PCL hyperarousal symptom severity PP** | | | |  | Time: | β = 2.11(1.01)*, t* = 2.1, *p* = 0.04 |
|  |  |  |  |  | Group * Time: | β = -0.58(0.54)*, t* = -1.07, *p* = 0.29 |
|  | LIC | 14.12(4.55) | 11.86(5.94) | .16 |  |  |
|  | HIC | 13.55(4.45) | 12.76(5.20) |  |  |  |
| **k. PCL negative cognition and mood symptom severity ITT** | | | |  | Time: | β = 2.32(0.88)*, t* = 2.64, *p* = 0.01 |
|  |  |  |  |  | Group * Time: | β = -0.05(0.55)*, t* = -0.1, *p* = 0.92 |
|  | LIC | 14.26(6.13) | 12.21(6.56) | .08 |  |  |
|  | HIC | 13.79(5.25) | 11.71(6.17) |  |  |  |
| **l. PCL negative cognition and mood symptom severity PP** | | | |  | Time: | β = 3.61(1.10), t = 3.28, *p* < .001 |
|  |  |  |  |  | Group * Time: | β = -0.44(0.59), *t* = -0.76, *p* = 0.45 |
|  | LIC | 13.79(5.25) | 11.21(6.22) | .31 |  |  |
|  | HIC | 14.26(6.13) | 13.10(6.02) |  |  |  |
